# Evaluation of the prophylactic use of mitomycin-C to inhibit haze formation after photorefractive keratectomy in high myopia: a prospective clinical study

**DOI:** 10.1186/1471-2415-4-12

**Published:** 2004-09-14

**Authors:** Hassan Hashemi, Seied Mohammad Reza Taheri, Akbar Fotouhi, Azita Kheiltash

**Affiliations:** 1 Farabi Eye Hospital, School of Medicine, Tehran University of Medical Sciences, Tehran, Iran; 2 Noor Vision Correction Center, Tehran, Iran; 3 Epidemiology and Biostatistics Department, School of Public Health, Tehran University of Medical Sciences, Tehran, Iran

## Abstract

**Background:**

To study the effect of prophylactic application of mitomycin-C on haze formation in photorefractive keratectomy (PRK) for high myopia.

**Methods:**

Fifty-four eyes of 28 myopic patients were enrolled in this prospective study. All eyes were operated by PRK followed by 0.02% mitomycin-C application for two minutes and washed with 20 ml normal saline afterwards. All eyes were examined thoroughly on the first 7 days and one month after surgery; 48 eyes (88.9%) at 3 and 6 months postoperatively. Hanna grading (in the scale of 0 to 4+) was used for assessment of corneal haze.

**Results:**

The mean spherical equivalent refraction (SE) was -7.08 diopters (D) ± 1.11 (SD) preoperatively. Six months after surgery, 37 eyes (77.1%) achieved an uncorrected visual acuity (UCVA) of 20/20 or better, all eyes had a UCVA of 20/40 or better and 45 (93.7%) eyes had an SE within ± 1.00D. One month postoperatively, 2 eyes (3.7%) had grade 0.5+ of haze, while at 3 and 6 months after surgery no visited eye had haze at all. All eyes had a best corrected visual acuity (BCVA) of 20/40 or better and there were no lost lines in BCVA by 6 months after surgery. In spatial frequencies of 6 and 12 cycles per degree contrast sensitivity had decreased immediately after PRK and it had increased 1.5 lines by the 6^th ^postoperative month compared to the preoperative data.

**Conclusions:**

The results show the efficacy of mitomycin-C in preventing corneal haze after treatment of high myopia with PRK. This method- PRK + mitomycin-C – can be considered an alternative treatment for myopic patients whose corneal thicknesses are inadequate for laser in situ keratomileusis (LASIK). However, the results should be confirmed in longer follow-ups.

## Background

In refractive surgery, patients with -5.00 diopters (D) myopia or more and corneal thicknesses less than 500 μm are not suitable candidates for laser in situ keratomileusis (LASIK) or conventional photorefractive keratectomy (PRK) due to the inadequate corneal thickness and risk of haze.[1-13] There are patients in which LASIK may seem possible, but the low pachymetry reading limits us to a small ablation zone, and as a result some post-LASIK complications such as glare and halos may occur. [14] If haze following PRK can be prevented in myopic eyes with a spherical equivalent (SE) over -5.00 D, obviously there would be less concern about the corneal thickness, ablation zone, and even the flap induced aberrations following Lasik. [15] Haze after PRK may result from the corneal wound healing process. In animal models, it has been shown that this process is probably initiated by keratocyte apoptosis and the subsequent over-proliferation of cells. [16] Collagen type IV alpha 3 is also an important factor in the development of corneal haze after PRK. [16] Mitomycin-C is an antibiotic with anti-metabolite effects that inhibit the proliferation of keratocytes,[17] but it has no effect on normal epithelial cells of the cornea. Mitomycin-C 0.02% has been used in the treatment of post-PRK haze. [18] There are also some reports on the prophylactic use of mitomycin-C to prevent haze following PRK in moderate to high myopia. [14,19]

In this prospective non-controlled clinical study we have assessed the prophylactic application of mitomycin-C on regression and haze in PRK performed on patients with high myopia (SE ≥ -5.00 D).

## Methods

### Study design and patient selection

Fifty-four eyes in 28 patients with a spherical equivalent refraction over -5.00 D were included in this prospective non-controlled clinical study. Since the residual stromal bed thickness under the flap after LASIK would be less than 250 μm, no eye had an appropriate corneal thickness for this procedure. In planning PRK, the post-ablation corneal thickness was calculated to be greater than 350 μm. Exclusion criteria in this study were systemic or ocular disease with the potential to interfere with the healing process of the cornea, such as collagenosis, diabetes, dry-eye syndrome, anterior or posterior uveitis, ectatic diseases such as keratoconus, and also corneal dystrophy or degeneration, glaucoma, retinal diseases, lens opacity, history of severe ocular trauma, and previous ocular surgery. The study was approved by the Research and Ethics Committee of the Noor Vision Correction Center. Before the operation, the nature of the procedures, their results and complications were thoroughly explained to all patients and they were asked to read and sign a formal informed consent in their native language.

### Procedure

All treatments were performed between April and October 2002. All eyes underwent the standard PRK procedure by two surgeons with extensive experiences with PRK. Patients received topical anesthesia without systemic sedation. Pre-incision of the corneal epithelium was made using a microtrephine with an 8 mm diameter and 70 μm deep calibrated blade (Janach, J 2900S). The epithelium was removed mechanically with a hocky knife. Then, the ablation was performed using a Technolas 217-C excimer laser (Bausch and Lomb, CA, USA). In all treatments, the overall ablation diameter was 8.4 to 8.9 mm and consisted of a central optical zone of 5.5 to 6.0 mm respectively by a 2.9 mm transition zone. Considering our previous experiences, eyes receiving mitomycin-C were intentionally under-corrected by 5 % compared with LASIK nomogram.

Immediately after laser ablation, a single topical application of mitomycin-C 0.02% (0.2 mg/ml) diluted in balanced salt solution was instilled in each eye with a weck sponge placed over the ablated stroma for 2 minutes. The corneal surface and the entire conjunctiva were then vigorously irrigated with 20 ml cold normal saline to remove the residual mitomycin-C. At the end of the procedure, a bandage contact lens was applied which was removed after three days.

After surgery, patients were instructed to take an analgesic (diclofenac sodium) every 8 hours and all eyes received artificial tears and flourometholone eye drop every four hours for 2 weeks, and chloramphenicol eye drop every 6 hours for 3 days. During the next two weeks, all patients were treated with flourometholone and artificial tears every 6 hours. These two eye drops were used every 8 and 12 hours during the second and third postoperative months, respectively, and were then discontinued. All patients were instructed to wear sunglasses in direct sunlight for 3 months.

The preoperative visit included uncorrected visual acuity (UCVA), best-corrected visual acuity (BCVA), manifest, subjective, and cycloplegic refractions, slit-lamp exams, applanation tonometry, corneal topography, ultrasonic pachymetry, keratometry, indirect funduscopy, and contrast sensitivity with and without glare. UCVA and BCVA were tested with the Snellen chart. On the first seven days after surgery, all patients were examined with a slit lamp and the area of the epithelial defect was measured with its ruler to identify the time of complete reepithelialization. On the 7^th ^and 14^th ^days after surgery we measured the UCVA, BCVA, and the manifest, subjective, and cycloplegic refraction. At the 1^st^, 2^nd^, and 6^th ^postoperative months, exams and measurements included UCVA, BCVA, manifest, subjective, and cycloplegic refraction, slit-lamp exams, applanation tonometry, ultrasonic pachymetry, keratometry, topography, and contrast sensitivity with and without glare. For evaluation of haze we used Hanna's grading scale from 0 (no haze) to 4+ (dense white corneal haze). Contrast sensitivity was tested by Vector Vision CSV-1000 (Vector Vision, Dayton, OH) in spatial frequencies of 6 (B) and 12 (C) cycles per degree. [20] The manufacturer's recommended testing procedures were followed. Absolute values of log contrast sensitivity were obtained for each patient and spatial frequency, and means and standard deviations were calculated. Data were then expressed in the notation of normalized log contrast sensitivity values. [21]

### Statistical analysis

Repeated measures analysis of variance was used to assess changes over time after surgery. For all statistical tests, the significance level was considered 0.05.

## Results

Of the 28 study group patients, 3 did not show up on the 3^rd ^and 6^th ^months visits (48 eyes out of 54 were visited; 88.9%). The mean age was 29.3 years (range, 20 to 45 years). The mean preoperative SE was -7.08 D ± 1.11 (SD) (range, -9.88 to -5.00D). The mean preoperative corneal thickness was 488.6 μm ± 11.9 (SD). At the first, 3^rd^, and 6^th ^month after surgery, the mean corneal thickness was 357 μm ± 30 (SD), 373 μm ± 27 (SD), and 380 μm ± 28 (SD), respectively. Thirty-three eyes (61.1 %) had a preoperative BCVA of 20/20 or better, and 51 eyes (94.4 %) had a BCVA of 20/25 or better. Complete reepithelialization was seen in no eye by the 2^nd ^postoperative day but in all eyes by the 6^th ^day. The epithelial defect had resolved in 53.7 % of the eyes by the 3rd day, and in 92.6 % by the 4th postoperative day.

**Figure 1:**
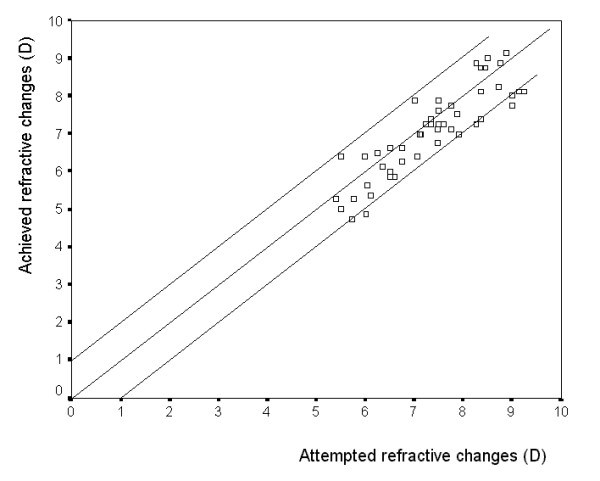
Scatter plots of attempted refractive changes versus the achieved refractive changes at 6 months after surgery (D = diopter).

**Figure 2:**
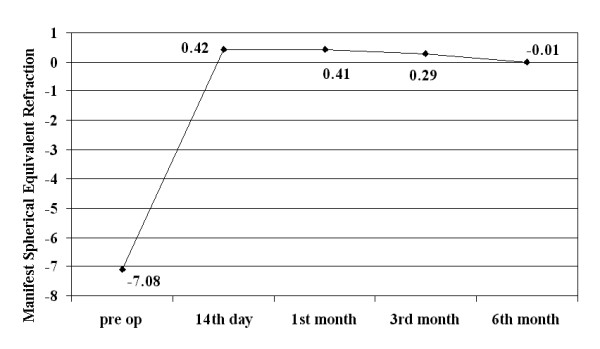
Mean manifest spherical equivalent refraction changes (diopter) over time.

Figure 1 shows the refractive results in this study group. At the 3^rd ^postoperative month, 33 eyes (68.7 %) were within ± 0.5 D of emmetropia (SE) and 43 eyes (89.6 %) within ± 1.00D (SE). At the 6^th ^postoperative month 39 eyes (81.3 %) were within ± 0.5D of emmetropia and 45 eyes (93.7 %) within ± 1.00D. Figure 2 shows the refractive changes after surgery during these six months. The refraction of patients had already become stable one month after surgery.

At the 3^rd ^postoperative month 32 eyes (66.7 %) had a UCVA of 20/20 or better and 47 (97.7 %) of 20/40 or better. All eyes had a BCVA of 20/40 or better and there were no lost lines in BCVA. On the 6^th ^month visit, 37 eyes (77.1 %) had a UCVA of 20/20 or better and 48 (100%) of 20/40 or better. All eyes had a UCVA of 20/40 or better. On the first month follow-up visit, 2 eyes (3.7%) had +0.5 haze, but all had 0 haze on the 3^rd ^and 6^th ^month visits.

**Figure 3:**
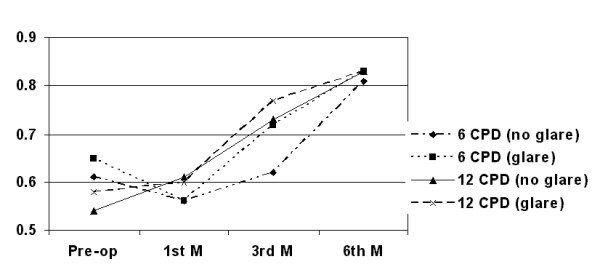
The changes of contrast sensitivity in spatial frequencies of 6 and 12 cycles per degree with and without glare over time (CPD = cycles per degree; M = month; D = diopter).

Figure 3 shows the changes of contrast sensitivity in spatial frequencies of 6 and 12 cycles per degree (CPD) with and without glare over time. In the spatial frequency of 6 CPD, contrast sensitivity had decreased immediately after PRK and it had increased by the 6^th ^postoperative month compared to preoperative data while in the spatial frequency of 12 CPD, it had increased over time after surgery (P < 0.001).

In our study, no complications such as eccentric ablation, delayed reepithelialization, persistent epithelial defect, or microbial keratitis have been observed.

## Discussion

PRK in high myopia remains a challenge due to its complications of haze and regression reported in previous experiments, and also the success rate of LASIK in these patients. [19] Yet, LASIK cannot be performed in some patients with an insufficient corneal thickness resulting in an undesirable residual stromal bed, or smaller ablation zones had to be applied to correct the refractive error completely. Such a small ablation zone can lead to visual inconveniences such as impaired night vision when the pupil dilates, halos, blurred vision, and ghost images. [15,22] If PRK plus mitomycin-C safety and predictability can be verified, larger ablation zones can be used, therefore the above complications will practically be avoided. On the other hand, correction of higher refractive errors will be possible.

This study was carried out to evaluate the safety and efficacy of PRK along with a 2-minute application of mitomycin-C 0.02% (0.2 mg/ml) on the exposed stromal bed after ablation was performed. Mitomycin-C is an antimetabolite and antibiotic drug. It is mostly used systemically in cancer chemotherapy. It has been used in ophthalmology in cases of intraepithelial neoplasms of the cornea and conjunctiva, ocular pemphygoid, and following surgical treatment of glaucoma and pterygium. Mitomycin-C has cytotoxic effects through inhibiting DNA synthesis. The logic behind using mitomycin-C is that the topical application of the drug on the cornea can inhibit subepithelial fibrosis through preventing the proliferation of stromal keratocytes, while the main causes of regression and haze are overacitvity and proliferation of stromal keratocytes following laser ablation. [19] The effects of mitomycin-C 0.02% in preventing haze has been shown by Talamo et al [22], and Xu et al [23] in experimental models. In a study by Majmudar et al, it was concluded that the application of mitomycin-C can remove haze following PRK and radial keratectomy (RK). [18] The usefulness of PRK with mitomycin-C (0.2 mg/ml) for preventing haze in high myopia was reported by Carones et al. [19] In this study we used the same concentration of 0.2 mg/ml to perform PRK + mitomycin-C in high myopia; considering the different climate and races in this Middle Eastern region. It seems that the problems of haze and regression after PRK are more prominent in people of this region. [24] In addition, the inclusion criteria aimed at patients who were at a relatively high risk of haze formation after PRK. Among factors determining the ablation depth, an important predictor of haze, correction and ablation zone size are more important. A 6.0 mm optical zone and a minimum of 5.00 diopters of correction ethically and practically limited our study to those patients who were at a high risk of developing haze with PRK.

No immediate toxic effects such as conjunctival chemosis or any delay or irregularity in re-epithelialization were seen. In only one case, after primary reepithelialization a large epithelial defect was seen on the fourth day (one day after complete epithelialization and contact lens removal), which healed in three days by applying a bandage contact lens. No complications such as corneal edema, melting, and perforation were observed in follow-up visits up to six months. Therefore, the topical application of mitomycin-C of the aforementioned concentration and duration was safe in our series. Yet, a longer follow-up is required to assess the long-term complications of mitomycin-C, and its careful application is recommended before long-term complications can be ruled out.

According to our previous experiences, we planned a 5% under-correction compared to our usual nomogram for LASIK. As a result of this modification, patients were close to emmetropia with a low variability on their 6^th ^month visit. In the report by Carones, patients had a hyperopic shift of +0.5D by the sixth month; this is while our patients had a myopic shift of 0.43D.

The UCVA at six months after surgery was 20/40 or better in 100% and 20/20 or better in 77.1% of patients. The refractive and visual results of this study are much better compared to reports concerning the same amount of correction with LASIK, [25,26] PRK, or LASEK [27,28].

Considering the fact that haze influences BCVA, patients' BCVA is of great importance. In this study, no eyes lost any lines of BCVA by six months after surgery compared to the preoperative BCVA. None of the eyes had any grade of haze 3 or 6 months after surgery, which shows the efficacy of prophylactic application of mitomycin-C in preventing haze following PRK for patients with myopia over -5.00D. In addition, an average postoperative improvement in contrast sensitivity of 1.5 lines points to the safety and efficacy of the procedure in the quality of vision.

In conclusion, using mitomycin-C in PRK for myopia greater than -5.00D seems safe and effective, and can reduce haze formation after surgery; therefore it can be considered a suitable alternative for patients with myopia greater than -5.00 D whose corneas lack an appropriate thickness to perform LASIK with a desirable optical zone. With this method, vision can be corrected with a better quality of vision regarding contrast sensitivity.

## Competing interests

None declared.

## Authors' contributions

HH, SMRT, AF and AK participated in the design of the study and the preparation of the manuscript. HH carried out all operations. SMRT visited patients in the follow up visits. AF and AK participated in the statistical analysis of the study. All authors have read and approved the final manuscript.

## Pre-publication history

The pre-publication history for this paper can be accessed here:
